# Prospects for use of bioengineered tissue from stem cells in gynecology

**DOI:** 10.1111/aogs.14621

**Published:** 2023-07-10

**Authors:** Mats Brännström, Mats Hellström

**Affiliations:** ^1^ Department of Obstetrics and Gynecology, Institute of Clinical Sciences, Sahlgrenska Academy University of Gothenburg Gothenburg Sweden

Bioengineering is a process in which an organ/tissue is created from stem cells and a scaffold; this process often includes a bioreactor. This principle has been explored by preclinical and human experimental research for a wide range of organs/tissues of the human body, including liver, kidney and heart. The application of bioengineering in gynecology would be to repair/replace a defect tissue/organ or to insert an organ/tissue that is missing. In this context, the specific organs and tissues of interest are the vagina, bladder, urethra, uterus, ovary, as well as supporting tissues of the pelvic floor. The Fallopian tube is less interesting, since oviductal defects with tubal‐factor infertility can be bypassed by in vitro fertilization. Bioengineering research in gynecology consists of several preclinical studies in animal models but only a few human pilot studies. In this editorial we address the hurdles related to clinical translation and outline a timeline for possible clinical application.

A major objective of the bioengineering principle is to bypass the impediments of organ/tissue shortage by using a biomaterial (also called scaffold) which is colonized by the patient's own cells to generate a patient‐specific grafting material. Since the organ/tissue is created by the patient's own stem cells, there is no need for immunosuppression to prevent rejection, as in allogeneic transplantation of solid organs or vascularized composite tissues.

There are two important structural components in the process of bioengineering, the scaffold and the cells to recellularize the scaffold with. The scaffold should be organ/tissue‐like in its three‐dimensional (3D) appearance and should provide structural benefits for the recellularized cells so that an appropriate construct can match the therapeutic requirements. The scaffold can be made from a synthetic substance or constructed from a biological material, typically an extracellular matrix‐component, with the possibility to build a complex scaffold by 3D printing. A more common approach today is to use a decellularized scaffold made from an organ/tissue obtained from a body after death, but in contrast to organ procurement from a deceased donor (multi‐organ donor), the limit to harvest the organ before (deceased brain‐dead donor) or within minutes after (deceased cardiac‐dead donor) cardiac arrest does not exist, since the organ to be decellularized may be procured several hours after termination of circulation.

Decellularization is carried out by a combination of physical and chemical steps to create a scaffold which is free from immunoreactive cellular components, including DNA, while leaving the tissue‐specific extracellular matrix preserved in its original 3D architecture and with remaining patent vascular conduits. The recellularization can be performed with pluripotent stem cells, which would be directed to the organ‐specific lineage by the scaffold‐specific extracellular matrix and remaining growth/differentiation factors. Alternatively, lineage‐specific stem cells can be used, ideally administered spatially specific to the different locations of the tissue/organ in a sequential order for recellularization to occur in vitro. Needless to say, a large organ with several different subcompartments and complex vascularity present marked hurdles during recellularization compared with a simple thin tissue of only a few cell types. Prior to recellularization, the stem cells have to be grown and expanded in culture so that large numbers (hundreds of millions/billions) of cells can be added to the scaffold for further creation of the organ/tissue in vitro using an incubator as a bioreactor.

There have been human trials of some bioengineered tissues/organs of relevance to gynecology, including the vagina, urethra and bladder, with all studies conducted by a research team in Wake Forest, USA. The results of the three studies, published 2006–2014, are reviewed below. Notably, there have not been any confirmatory studies from this or other research groups, which could indicate difficulties replicating the results, or that other studies are prioritized.

Use of a bioengineered vagina is applicable in congenital vaginal‐absence such as in the Mayer–Rokitansky–Küster–Hauser syndrome (MRKHs), after surgery for primary vaginal cancer and after excenteration surgery in cases of recurrence of gynecological cancer. In a small study, four young women with MRKHs underwent insertion of bioengineered vaginal organs.^1^ Vulvar biopsies (1 × 1 cm) were acquired to culture and expand autologous muscle cells and epithelial cells, which after expansion in vitro were seeded onto a biodegradable scaffold made by non‐human intestinal submocosa. The vagina‐like scaffold (around 8 cm long) was further cultured in a bioreactor for 5 weeks and then surgically implanted by a perineal approach. Follow‐up for 8 years after surgery showed normal vaginal tissue on histology of biopsies and sexual function was normal, as evaluated by validated questionnaires.^1^

The concept of bioengineered bladder was tested in seven women with myelomeningocele and poorly compliant bladders.^2^ A bladder biopsy was used to obtain patient‐specific urothelial and muscle cells, which were grown and expanded in vitro followed by seeding them onto a biodegradable composite or a decellularized bladder scaffold, with an area of around 100 cm^2^. After 7 weeks of development in a bioreactor, the tissue was used for bladder reconstruction and implanted with an omental flap. Functional tests from 2 years after surgery showed increased volume and compliance.^2^

The female urethra can be affected by congenital malformations or injury/loss at surgery, and replacement/repair may be needed. A bioengineered urethral segment would have its most common use in reconstructive surgery after urethral strictures in males, and much less frequently in women. One study evaluated bioengineered urethral segments in five boys with urethral strictures and with cells grown and expanded from urethral biopsies. The cells were seeded on synthetic biodegradable scaffolds and grown in vitro for 5 weeks.^3^ Tissue architecture by histology was normal 3 months after insertion of the 4‐ to 6‐cm‐long bioengineered grafts and follow‐up to 6 years did not show any signs of restenosis. Given the short length of the female urethra, this principle could be used in the female, but preserved function of the internal and external urethral sphincter muscle would be a prerequisite to acquire continence. Alternatively, these muscles could be created by bioengineering principles, as explored in animal models.^4^

Pelvic floor injuries are frequent after vaginal deliveries and can cause pelvic organ prolapse and urinary incontinence. There have been several attempts to repair muscular/connective tissue defects by synthetic meshes, but with serious, long‐term adverse side effects such as erosion, infection and chronic pain. Animal experiments have been conducted to test bioengineering principles to acquire pelvic floor muscle tissue for this purpose, as evaluated from muscle cells by a Danish group^5^ or from mesenchymal stem cells by an Australian group.^6^

Bioengineering of the ovary and uterus could be applied in women of fertile age, with defects/absence of these organs and a fertility desire. Our group, being active in preclinical and clinical research on uterus transplantation for many years, started an animal‐based research project on uterus and ovarian bioengineering around 10 years ago.

Bioengineered uterine tissue could be used for the repair of uterine defects or corrections of malformations. A complete bioengineered uterus could replace uterus transplantation as an infertility treatment for absolute uterine factor infertility. So far, segments of bioengineered uterine tissue derived from decellularized uterine scaffold with recellularization by primary uterus cells and mesenchymal stem cells have been used to restore fertility after full‐thickness uterine tissue replacement in rats.^7^ A similar concept was also evaluated in rabbits after a biodegradable synthetic scaffold with autologous uterus cells was used to replace an extensive part of the rabbit uterus horn.^8^ These studies provide proof‐of‐concept but, needless to say, bioengineering of a complete uterus, including vascular conduits, will be a challenging procedure and such development will most likely take many years.

Fertility preservation in young female cancer patients by ovarian cortex cryopreservation is clinically established and numerous births have been reported after avascular retransplantation of cortical tissue after successful cancer treatment. However, retransplantation after cryopreservation in women with disseminated hematopoetic cancers, such as leukemia, has not been performed due to the high risk of reintroducing malignant cells. Preclinical work is conducted to construct an ovarian scaffold with non‐follicular supporting cells using bioengineering principles.^9^ The aim would be to seed patient‐derived small follicles that are free from cancer cells, which may be present in the extrafollicular ovarian stroma of cryopreserved tissue, onto these scaffolds and subsequently develop the ovarian‐like construct further in a bioreactor,^10^ before a fertility restoration transplantation.

In the beginning of the era of organ bioengineering research, the general views on when to reach a clinical application were optimistic. However, with increasing knowledge and several identified hurdles, we approach the task with more humility. For example, translation of principles from successful rodent studies to larger and more clinically relevant animal models have exposed new challenges related to the recellularization process in larger bioengineered constructs.

Furthermore, the degradation profile after engraftment changes with increasing construct size, which will need to be adapted to suit the required support during organ/tissue regeneration in vivo. Additionally, the revascularization of sizable, bioengineered grafts becomes more challenging and non‐optimal results may jeopardize the therapeutic outcomes. Hence, successful larger bioengineered grafts will most likely also include the reconstruction of a vascular network within the graft itself that can be connected to the host's vasculature through anastomoses during engraftment to prevent graft ischemia.

Progress in stem cell research will further assist the progress in reproductive bioengineering research, since the selection of cells used for the recellularization process can be further optimized.

Although initial clinical trials of human bioengineered tissues have commenced, especially for different bone and skin regeneration regimens, it will surely take many years until this principle is in full clinical use. Concerning its use in gynecology, we predict that bioengineered tissue may be tested in larger clinical trials within 10 years, but it will most likely take longer before whole organ bioengineering principles can be clinically tested in gynecology.
